# A multilayer network analysis of Alzheimer's disease pathogenesis: Roles for p‐tau, synaptic peptides, and physical activity

**DOI:** 10.1002/alz.14286

**Published:** 2024-10-12

**Authors:** Andrea A. Jones, Alfredo Ramos‐Miguel, Kristina M. Gicas, Vladislav A. Petyuk, Sue E. Leurgans, Philip L. De Jager, Julie A. Schneider, David A. Bennett, William G. Honer, Kaitlin B. Casaletto

**Affiliations:** ^1^ Division of Neurology Department of Medicine University of British Columbia Vancouver British Columbia Canada; ^2^ Department of Pharmacology Centro de Investigación Biomédica en Red de Salud Mental (CIBERSAM) University of Basque Country (EHU/UPV) Leioa Spain; ^3^ Biocruces Bizkaia Health Research Institute Barakaldo Spain; ^4^ Department of Psychology University of the Fraser Valley Abbotsford British Columbia Canada; ^5^ Biological Sciences Division Pacific Northwest National Laboratory Richland Washington USA; ^6^ Rush Alzheimer's Disease Center Rush University Chicago Illinois USA; ^7^ Department of Neurology and The Taub Institute for the Study of Alzheimer's Disease and the Aging Brain Center for Translational and Computational Neuroimmunology Columbia University Medical Center New York New York USA; ^8^ Department of Psychiatry University of British Columbia Vancouver British Columbia Canada; ^9^ BC Mental Health and Substance Use Services Research Institute Vancouver British Columbia Canada; ^10^ Department of Neurology Memory and Aging Center University of California San Francisco California USA

**Keywords:** aging, Alzheimer's disease, physical activity, *post mortem* brain, presynaptic proteins, proteomics, synaptopathy

## Abstract

**INTRODUCTION:**

In the aging brain, cognitive abilities emerge from the coordination of complex pathways arising from a balance between protective lifestyle and environmental factors and accumulation of neuropathologies.

**METHODS:**

As part of the Rush Memory and Aging Project (*n* = 440), we measured accelerometer‐based actigraphy, cognitive performance, and after brain autopsy, selected reaction monitoring mass spectrometry. Multilevel network analysis was used to examine the relationships among the molecular machinery of vesicular neurotransmission, Alzheimer's disease (AD) neuropathology, cognition, and late‐life physical activity.

**RESULTS:**

Synaptic peptides involved in neuronal secretory function were the most influential contributors to the multilayer network, reflecting the complex interdependencies among AD pathology, synaptic processes, and late‐life cognition. Older adults with lower physical activity evidenced stronger adverse relationships among phosphorylated tau peptides, markers of synaptic integrity, and tangle pathology.

**DISCUSSION:**

Network‐based approaches simultaneously model interdependent biological processes and advance understanding of the role of physical activity in age‐associated cognitive impairment.

**Highlights:**

- Network‐based approaches simultaneously model interdependent biological processes.
- Secretory synaptic peptides were influential contributors to the multilayer network.
- Older adults with lower physical activity had adverse relationships among pathology.
- There was interdependence among phosphorylated tau, synaptic integrity, and tangles.
- Network methods elucidate the role of physical activity in cognitive impairment.

## 1 BACKGROUND

The prevalence and impact of dementias are increasing across the global aging population, affecting > 57 million people worldwide.^1^ In the aging brain, cognitive abilities emerge from the coordination of complex brain systems^2^ threatened by accumulation of neuropathologies over the life course. In Alzheimer's disease (AD), the most common cause of dementia, neuropathological hallmarks include accrual of amyloid beta (Aβ) as insoluble plaques, aggregation of hyperphosphorylated tau as neurofibrillary tangles, and subsequent neurodegeneration.^3^ The relationships between neuropathology and synaptic functioning underlying cognition are complex. Pathological features may create both local and global toxicities harming synaptic terminal machinery, leading to synapse dysfunction and loss.^4^, ^5^ A complementary model suggests early aberrant synaptic functioning may accelerate the development of pathology by promoting tau hyperphosphorylation.^6^, ^7^ In contrast, greater density of functionally active synapses indexed by synaptic morphology, protein levels, or functional imaging is associated with resilience against cognitive decline in the context of AD pathophysiology.^8^, ^9^, ^10^, ^11^, ^12^ Taken together, a deeper understanding of the interplay among the diversity of synaptic processes alongside progressive neuropathology and their relationship with clinical outcomes in humans is needed to fundamentally understand AD dementia risk.

Decades of animal, clinical, and epidemiological studies demonstrate that synaptic functioning and cognition are bolstered by enriching late‐life lifestyle activities, such as physical activity, while relative neglect may account for ≈ 30% to 40% of dementia cases.^13^, ^14^ Physical activity is associated with decreased risk of AD dementia,^15^, ^16^ improved cognition,^17^, ^18^ and preserved brain structure and function in populations over age 65,^19^, ^20^, ^21^ relationships showing particularly strong effects in the presence of AD pathology.^22^, ^23^, ^24^ Physical activity is consistently reported to enhance synaptogenesis and improve behavioral outcomes in animal studies, including models of injury.^25^, ^26^

Enriching our understanding of the effects of physical activity on cellular pathophysiology in humans may facilitate our understanding of cognitive resilience to AD pathogenesis and identify the highest yield targets for intervention and prevention. Investigation of the functional properties of synapses is a strategy that can be applied to human *post mortem* samples.^12^, ^24^, ^27^, ^28^ Functional vesicular neurotransmission requires the formation of a SNARE (soluble N‐ethylmaleimide‐sensitive factor attachment protein receptor) protein complex, comprised of syntaxin‐1 (STX1), synaptosome‐associated protein 25 (SNAP25), and vesicle‐associated membrane protein (VAMP or synaptobrevin). SNARE complex formation and presynaptic release of neurotransmitter requires multiple protein partners, forming the SNARE interactome.^12^, ^29^

Similar to other areas of medicine, analysis of the interactions among molecular events such as neurotransmission, with cellular pathology and lifestyle at multiple, hierarchical levels is advanced by application of complexity science.^30^, ^31^, ^32^ Approaches such as network analysis can examine the interplay between biological and lifestyle factors driving disease. The relationships between these factors may be indirect, interdependent, or non‐linear, represented as nodes forming a network. Analytics of local and global network properties may reveal critical factors influencing the dynamics of the brain–environment system. Multilayer network analysis was developed to examine interrelationships across high‐dimensional data.^33^, ^34^, ^35^ This modeling strategy enables assessment of relationships within and across scales, such as molecular and cellular biological systems. Multilayer network approaches have not yet been applied to questions regarding larger scale proteomics, neuropathology, and modifiable lifestyle factors to understand brain aging.

As part of the Rush Memory and Aging Project (MAP), we collected objective activity levels with accelerometer‐based actigraphy along with cognitive measures of older adults who consented to *post mortem* brain autopsy providing tissue for immunohistochemistry and selected reaction monitoring mass spectrometry analysis. Previous MAP studies found that actigraphy‐based physical activity was associated with reduced incidence of AD, improved cognitive trajectories,^15^ and higher levels of individual synaptic proteins in brain tissue using low throughput methods (enzyme‐linked immunosorbent assay [ELISA]).^27^ The present analyses aim to apply a multilayer network analysis approach to examine the relationships among 48 SNARE interactome (referred to here as “synaptic”) peptides, AD neuropathology, and cognition in a longitudinal community‐based sample of older adults. The second aim is to investigate the cellular mechanisms of physical activity by testing how late‐life physical activity affects the synaptic‐pathology multilayer network structure. We expect that the multilayer network analytic approach will provide a more complete representation of the interdependencies between synaptic and AD pathophysiology, and help identify influential nodes within the aging brain system.

## 2 METHODS

### 2.1 Participants

Samples for the present study were obtained from brain autopsies of participants in the Rush MAP.^36^ This is a longitudinal, community‐based study of cognition in participants aged ≥ 65 years, free from known dementia at enrolment. Participants sign an informed consent and Anatomical Gift Act, as well as a repository consent allowing data and specimens to be shared for research studies. The overall follow‐up rate of MAP participants is 95%, with an autopsy rate of > 80%. A proteomic study was carried out using dorsolateral prefrontal cortex (DLPFC) samples from *n* = 659 participants in MAP; the present analyses include *n* = 440 participants with available assessments of physical activity.

### 2.2 Physical activity assessment

An activity monitor was worn on the non‐dominant wrist to measure rest/activity continuously (24 hours a day) for up to 10 days (Actical; Mini Mitter). Activity counts were extracted from each 15‐second epoch yielding 5760 data points/day. For analyses, incomplete days of data were excluded, based on inspection of recordings with an automated program flagging extreme average daily counts: ≈ 0/day or > 500/day. Only participants with valid data for one or more days were included in analyses. During the 10‐day monitoring period both weekday and weekend time were captured. Daily physical activity summarized both exercise and non‐exercise activities, and was calculated for full days of data as the average sum of all daily activity counts for each 15‐second epoch.^15^ Participants completed a mean of 3.5 (standard deviation [SD] = 2.4) visits with physical activity assessments; 73% completed two or more visits. Patients with adequate actigraphy measures were older (mean, SD: 90.6, 6.2 vs. 88.8, 6.6, *P* < 0.001) and less likely to be male (mean, SD 0.27, 0.42 vs. 0.35, 0.48, *P* = 0.003). Overall late‐life physical activity levels were examined by averaging daily activity counts across all available visits per participant.

**RESEARCH IN CONTEXT**

1. **Systematic review**: The authors reviewed the literature using traditional sources. While decades of animal, clinical, and epidemiological studies demonstrate that physical activity bolsters synaptic functioning and cognition and reduces incidence of Alzheimer's disease (AD), a deeper understanding of the interplay among the diversity of synaptic processes, progressive neuropathology, and clinical outcomes is needed to fundamentally understand AD risk.
2. **Interpretation**: Our findings led to an integrated model of the complex interdependencies among AD pathology, synaptic processes, physical activity, and cognition. This article reconciles divergent areas of inquiry, including studies of proteomics, neuropathology, and cognitive resilience.
3. **Future directions**: Our novel approach highlights the utility of advanced network approaches for high‐dimensional biobehavioral data, setting a framework for further study, including (1) molecular pathogenesis of interdependence among phosphorylated tau, neuronal secretion, and tangle pathology; (2) mechanistic studies of clinically relevant neuronal secretion targets; and (3) network studies of high‐yield lifestyle interventions for AD prevention and treatment.

### 2.3 Cognitive assessment

A standardized, annual neuropsychological assessment involved 21 tests, 19 of which were summarized as episodic memory, semantic memory, working memory, visuospatial ability, and perceptual speed completed at the study visit closet to death.^37^ The Mini‐Mental State Examination was administered for description. An index of global cognition was calculated by first converting raw scores to *z* scores using the mean and SD at baseline, then taking an average.^38^ Participants were also assessed annually for dementia, mild cognitive impairment, and no cognitive impairment as described.^39^ A final diagnosis was made by review of select data across all years by an experienced neurologist blinded to all *post mortem* data. Apolipoprotein E (*APOE*) genotype was determined as previously reported.^40^

### 2.4 Neuropathologic assessment

A standardized neuropathological examination was carried out as previously described.^41^ Briefly, after being weighed, brains were cut coronally into 1‐cm thick slabs, immersed in 4% paraformaldehyde for 48 to 72 hours, and placed in 2% dimethylsulfoxide/2% glycerol in phosphate‐buffered saline for storage. Examination was completed by experienced examiners blinded to demographic and clinical information. The assessment induced counts of neuritic and diffuse plaques and neurofibrillary tangles based on silver stain; these were also used to inform Consortium to Establish a Registry for Alzheimer's Disease and Braak stage, which were combined to an National Institute on Aging/Reagan pathologic diagnosis. Aβ load and tau tangle density were based on immunostains. The assessment also included Lewy body disease, hippocampal sclerosis, and atherosclerosis.

### 2.5 Targeted liquid chromatography selected reaction monitoring proteomics

Samples for proteomic analysis were obtained from the DLPFC, approximating Brodmann's area (BA) 46/9, and frozen at −80°C. With these samples, a liquid chromatography (LC) selected reaction monitoring (SRM) strategy was applied to quantify the relative amounts of 60 peptides representing SNARE complex interacting proteins, coded by 30 genes. Fourteen peptides assessed amyloid and tau proteins, the latter including separate measures of non‐phosphorylated and synonymous phosphorylated sequences.^12^, ^42^ Naming conventions used here, and descriptions of the design of peptide sequences, targets, and properties appear in Tables S1–S4 in supporting information. The LC‐SRM estimates target (light) peptide abundances relative to the spiked‐in reference (heavy) peptide standards. The light‐to‐heavy peptide ratios were log2‐transformed and centered at the median. Where the observed amounts of two peptides designed to detect the same synaptic protein were highly correlated (*n* = 12 pairs), a mean value was calculated. Peptides belonging to different splice variants of the same gene were studied separately. A total of 48 synaptic peptides, and 14 pathological peptides are represented as nodes in network analyses (Tables S1 and S3).

### 2.6 Cellular AD pathology

A Bielschowsky‐modified silver impregnation protocol assessed total count of neurofibrillary tangles, neuritic plaques, and diffuse plaques. We additionally assayed amyloid and phosphorylated tau (p‐tau) levels via immunohistochemistry. Aβ was immunolabeled with M00872 (1:100), which binds to Aβ species of both 1–40 and 1–42 lengths. A standardized immunostaining procedure was used to estimate amyloid deposition in the selected brain region.^43^ Percentage area occupied was estimated. Paired helical filament tau was labeled with AT8 (1:800), an antibody specific for phosphorylated tau. Quantification of tangle densities was performed using stereological image count analyses via Leica DMRBE microscope and computer (Millennia Mme; Micron Electronic Inc.) and Stereo Investigator software, version 8.0 (50% region sample with 150 µm × 150 µm counting frame at × ≈ 40 magnification). For all amyloid and tau metrics, estimated levels were quantified within the DLPFC to mirror the LC‐SRM assays. Values were scaled by dividing by the regional standard deviation.

### 2.7 Synaptic protein–protein interactions

A microplate‐based immunoprecipitation assay was used to quantify binary protein–protein interactions between the SNARE proteins syntaxin‐1, SNAP‐25, and VAMP as previously described.^44^ In this manner, incorporating functionally relevant synaptic targets previously shown to be associated with cognitive outcomes allows for a more comprehensive description of relevant synaptic processes in the multilayer network.^45^

### 2.8 Statistical analyses

#### 2.8.1 Synaptic peptide network and multilayer network estimation

Network estimation, visualization, assessment, and group comparisons were performed using R (R Core Team, 2017), specifically igraph, networktools, qgraph, bootnet, NetworkComparisonTest, signnet, and huge packages.

A multilayer network was also constructed to include four layers representing synaptic peptides, synaptic protein–protein interactions, AD pathological peptides, and AD cellular pathology. The synaptic peptide layer included 48 nodes representing the relative abundance of each peptide (Table S1). The synaptic protein–protein complex layer included four nodes representing binary SNARE protein–protein interactions (Table S2). The pathological peptide layer included 14 nodes representing Aβ, amyloid precursor peptides, and phosphorylated and non‐phosphorylated tau peptides (Table S3). The cellular pathology layer included five nodes representing diffuse and neuritic plaques, neurofibrillary tangles (Bielschowsky and immunostained), and amyloid (Table S4).

The multilayer network was constructed by estimating a Gaussian graphical model with L_1_‐regularization to limit Type 1 errors and improve estimation accuracy, sparsity, and interpretation.^46^, ^47^ The edges of this weighted and signed network represents conditional dependence relations and are partial correlation estimates between all pairs of nodes, controlling for all other variables in the network. Intra‐layer edges represent the pairwise conditional dependence between nodes within the same layer (e.g., interdependences between pairs of protein–protein interactions), while intra‐layer edges represent pairwise relationships across network layers. A non‐paranormal transformation was applied to the data prior to estimation to account for departures from multivariate normality.^48^ To avoid false positive findings, the graphical least absolute shrinkage and selection operator (LASSO)^46^ L_1_‐regularization technique was applied with extended Bayesian information criterion (EBIC) for model selection,^49^ using a hyperparameter of gamma = 0.5, which has been shown to yield parsimonious models with good sensitivity and specificity.^50^ Missing data were removed by pairwise deletion. The Fruchterman–Reingold algorithm was used to plot the network, placing related nodes closer together in space.^51^

Local and global network measures were estimated. Network topology was studied for evidence of scale‐free behavior, small world property, hub nodes, and structural equivalence among nodes.^52^, ^53^ Network scale‐free properties were assessed by testing whether the degree distributions followed a power‐law distribution using the weighted Kolmogorov–Smirnov test.^54^ This property is common among many biological networks, whereby a few important nodes have many edges (“hub” nodes), and a large number of nodes have few edges. This unifying paradigm across complex systems facilitates efficient communication and influence through the network, and resilience against perturbation. Diameter (the maximum number of steps separating any two nodes) was also calculated.

Centrality measures were calculated for each node to determine their role and importance in the network. The centrality measures included strength, closeness, and betweenness.^55^ Strength is the sum of the edge weights for each node and is a measure of how strongly connected a node is to other nodes. Closeness is the sum of the inverse shortest paths to all other nodes and is an estimate of efficiency and how strongly a peptide is indirectly associated with all other peptides in the network. Betweenness is the number of times the peptide lies on the shortest path between two other nodes, and thus represents its role as a gatekeeper between other pairs of peptides. Hub nodes are nodes with betweenness centrality greater than two standard deviations from the mean. Expected influence, a measure of a nodes’ relative importance, was also calculated for each node, and is the sum of all edges extending from a given node, taking into account both negative and positive edges.^56^ Global connectivity was estimated as the sum of all absolute edge‐weights, and reflects the degree of interdependence among nodes.

In a signed network, in addition to the number and position of negative edges, we can also consider the distribution of negative edges across the network, by considering higher‐order network structure. Structural balance theory suggests that node‐triad relationships are inclined to assume stable configurations, which can be indicated by a balanced triangle—such as two negative and one positive edge.^57^ Unbalanced triangles (e.g., one negative and two positive edges) can be sources of structural tension in the network, which indicates these relationships may be tenuous and potentially modifiable. The degree of global structural balance, a measure of node‐triad higher‐order network structure, was estimated as the proportion of balanced versus unbalanced triangles in the network and is thought to be reflective of potential network dynamics.^57^, ^58^ Higher structural balance indicates stability and potentially conservation of network structure.

#### 2.8.2 Synaptic peptide network module detection

As global network properties can emerge from local interactions, we next examined modules representing groups of highly interdependent nodes, and examined shared or distinct biological functional properties within and between modules. Many biological networks organize into modular structures, for improved efficiency and resilience.^59^ To better evaluate the local network structural features exhibited by the synaptic peptides alone, a separate network was estimated that included the 48 synaptic peptides only. Modules were detected by applying the Spinglass community detection algorithm, which accounts for both positive and negative edges to identify modules of nodes that have relatively dense positive (and sparse negative) connectivity within the module, compared to peptides belonging to different modules.^60^ This algorithm demonstrated good performance in small (<1000 nodes) networks.^61^ Network modularity (the strength of the division of a network into modules) and transitivity (or global clustering coefficient, the tendency of nodes to cluster together) were also measured. Relationships between module membership and subcellular localization, cell type, and peptide function were assessed by analysis of variance.

#### 2.8.3 Multilayer networks compared by late‐life physical activity

Multilayer networks of pathology and synaptic peptides were estimated and compared by participants’ level of late‐life physical activity: greater than (higher physical activity group; *n* = 220) and less than (lower physical activity group, *n* = 220) median daily activity counts as measured by an actigraphy device. As above, the nodes included synaptic peptide levels, synaptic protein–protein complexes, pathological peptide levels, and cellular pathology by immunohistochemistry. Group networks were compared using a bootstrapping procedure that reshuffled group membership and re‐estimated the networks 10,000 times.^62^ Group differences in network‐level features (global connectivity, module structure, and edge‐weights) and node‐level features (centrality) were compared to the permutation distribution. Holm–Bonferroni correction was applied to identify significant differences to account for multiple comparisons.

#### 2.8.4 Network‐informed analyses

Last, to better understand the multilayer network patterns, we tested a final regression model including the key node and modules that emerged, including p‐tau hub nodes, synaptic module with the highest network influence, and cellular tangle pathology alongside physical activity levels. The model examined how levels of physical activity (continuous daily counts) moderated the relationships among p‐tau and synaptic modules on tangle pathology burden levels. To do so, we entered three‐way interaction terms (physical activity x p‐tau synaptic module ∼ tangle pathology) for both hub p‐tau peptides identified.

## 3 RESULTS

### 3.1 Participants

Demographic variables and clinical assessments of participants appear in Table 1. Samples of DLPFC were drawn from a larger series reported previously (*n* = 1226)^12^ with the additional inclusion criteria of available physical activity data.

**TABLE 1:** Demographic and clinical characteristics of the sample.

Variable	(*n* = 440)
Demographic	
Female, no. (%)	323 (73.4)
Age at death, years (mean, SD)	90.6 (6.2)
Education, years (mean, SD)	14.6 (2.8)
*APOE* ε4 carriers, no. (%) (*n* = 427)	100 (23.4)
Average late‐life lifestyle factors	
Actigraphy: average daily counts (mean, SD)	1.94 (1.14)
Cognitive function proximate to death	
Clinical diagnosis, no. NCI:MCI:DEM	149:114:177
Global cognition *z* score (mean, SD)	−0.92 (1.14)
MMSE score (mean, SD)	21.2 (8.7)
Pathologic	
NIA/Reagan scale, no. 1:2:3:4	82:214:141:3
CERAD scale, no. 1:2:3:4	155:153:38:94
Braak stage, no. 0–II:III:IV:V‐VI	57:105:152:119:7
Amyloid beta middle frontal, area fraction (*n* = 439)	6.86 (6.30)
Tangles (AT8) middle frontal, density (*n* = 439)	2.43 (7.97)
Diffuse plaques middle frontal, density (*n* = 439)	15.70 (17.81)
Neuritic plaques middle frontal, density (*n* = 439)	10.69 (11.50)
Neurofibrillary tangles middle frontal, density (*n* = 439)	2.66 (7.17)
Atherosclerosis, no. 0:1:2:3	108:222:83:27
Lewy body disease, no. (%) (*n* = 428)	107 (25.0)
Hippocampal sclerosis, no. (%)	47 (10.7)

Abbreviations: *APOE*, apolipoprotein E; CERAD, Consortium to Establish a Registry for Alzheimer's Disease; DEM, dementia; MCI, mild cognitive impairment; MMSE, Mini‐Mental State Examination; NCI, no cognitive impairment; NIA, National Institute on Aging; SD, standard deviation.

### 3.2 Synaptic and multilayer peptide network characteristics

A signed, weighted, undirected synaptic peptide network was constructed from the final set of 48 peptides. The network was assessed for evidence of structural equivalence. We found relationships among peptides encoded by the same gene were sufficiently unique to consider each peptide as an independent node. This network is considered scale‐free, as the degree distribution follows a power‐law distribution (Kolmogorov–Smirnov 0.283, *P* = 0.724), and also demonstrates small world properties with a small diameter (0.041). These network properties represent an underlying unifying paradigm across biologically complex systems that facilitates rapid communication between nodes and resilience against perturbation.^63^, ^64^

**FIGURE 1:**
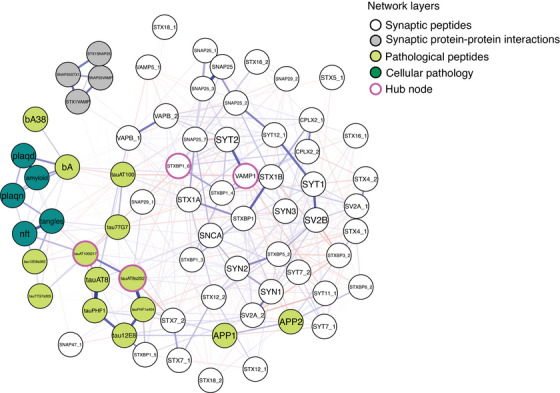
Multilayer network of synaptic peptides (white), synaptic protein–protein interactions (gray), pathological peptides (lime), and cellular pathology (teal). Hub nodes highlighted in pink. Edge weights represent the positive (blue) and negative (red) conditional dependencies between nodes. Fruchterman–Reingold layout whereby nodes closely positioned have stronger relationships.

A multilayer network was constructed to investigate relationships among layers representing synaptic peptides (as above), synaptic protein–protein interactions (measured via “capture” ELISA), pathological peptides, and cellular pathology detected by immunostaining for Aβ and p‐tau, as well as by Bielschowsky silver staining (Figure 1). This multilayer network is also considered scale free, as its degree distribution follows a power law (Kolmogorov–Smirnov 0.167, *P* = 0.996), and it also demonstrated small world properties with a small diameter (0.234). An important property of scale‐free networks is the presence of a few important hub nodes, as well as a large number of nodes with few connections, a structure which enables efficient communication and influence throughout the network.

Local and global network measures were estimated for the multilayer network. Centrality measures were calculated for each node of the multilayer network (Figure S1 in supporting information). Hub nodes are defined by betweenness centrality (the number of times the peptide lies on the shortest path between two other nodes, and thus represents its role as a gatekeeper between other pairs of peptides), specifically betweenness > 2 SDs from the mean. Hub nodes identified from the pathological peptides layer included tau_AT100_t217 and tau_AT8_s202, and within the synaptic peptides layer included STXBP1_6 and VAMP1. STXBP1_6 and the neighboring node STXBP1_4 is derived from the long splice variant of the *STXBP1* gene, also known as Munc18‐1‐a, and is enriched in GABAergic, inhibitory terminals.^65^

### 3.3 Modules within the synaptic peptide network

**FIGURE 2:**
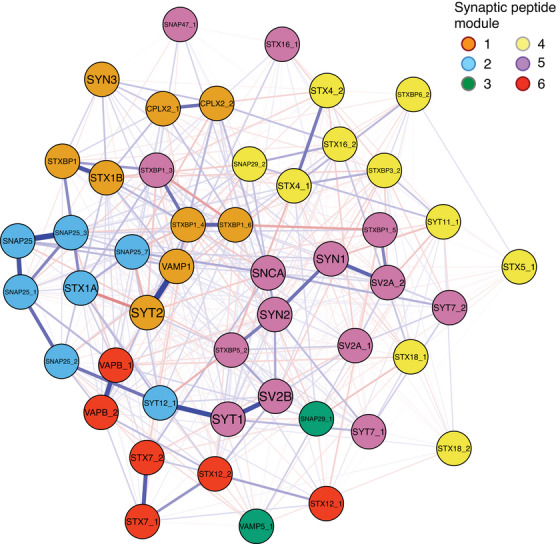
Synaptic peptide network with six modules identified: module 1 (orange), module 2 (blue), module 3 (green), module 4 (yellow), module 5 (purple), and module 6 (red). Edge weights represent the positive (blue) and negative (red) conditional dependencies between nodes. Fruchterman–Reingold layout whereby nodes closely positioned have stronger relationships.

**FIGURE 3:**
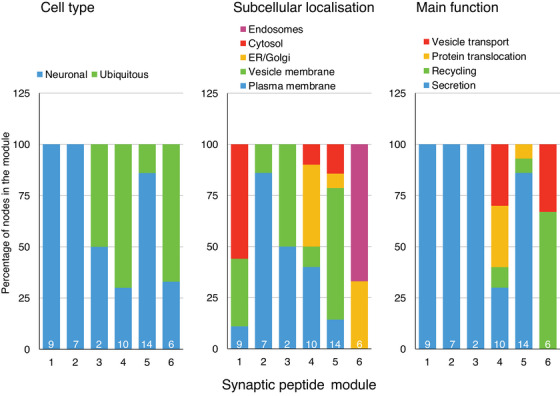
Stacked barplots of the proportion of synaptic peptides in each module by (left to right) cell type, subcellular localization, and primary function.

As global network properties can emerge from local interactions, we next examined the synaptic peptide network structure for modules that represent groups of individual peptide nodes, and examined the modules detected for shared or distinct biological functional properties (Tables S1–S4). Six synaptic modules were identified, with modest modularity (modularity = 0.216) and moderate transitivity (global clustering coefficient = 0.469), indicating a tendency of peptides to cluster together (Figure 2). Modules were comprised of 2 to 14 peptide nodes. The cell type, subcellular localization, and functional characteristics of each module are illustrated in Figure 3. Notably, peptide nodes in module 1 and 2 were exclusively neuronal, and exclusively involved with secretion. Peptides forming module 2 nodes were localized to the plasma or vesicular membranes, while a majority of module 1 peptides were localized to the cytosol. Both overlapping but distinct processes appear to be represented across the six synaptic modules.

**FIGURE 4:**
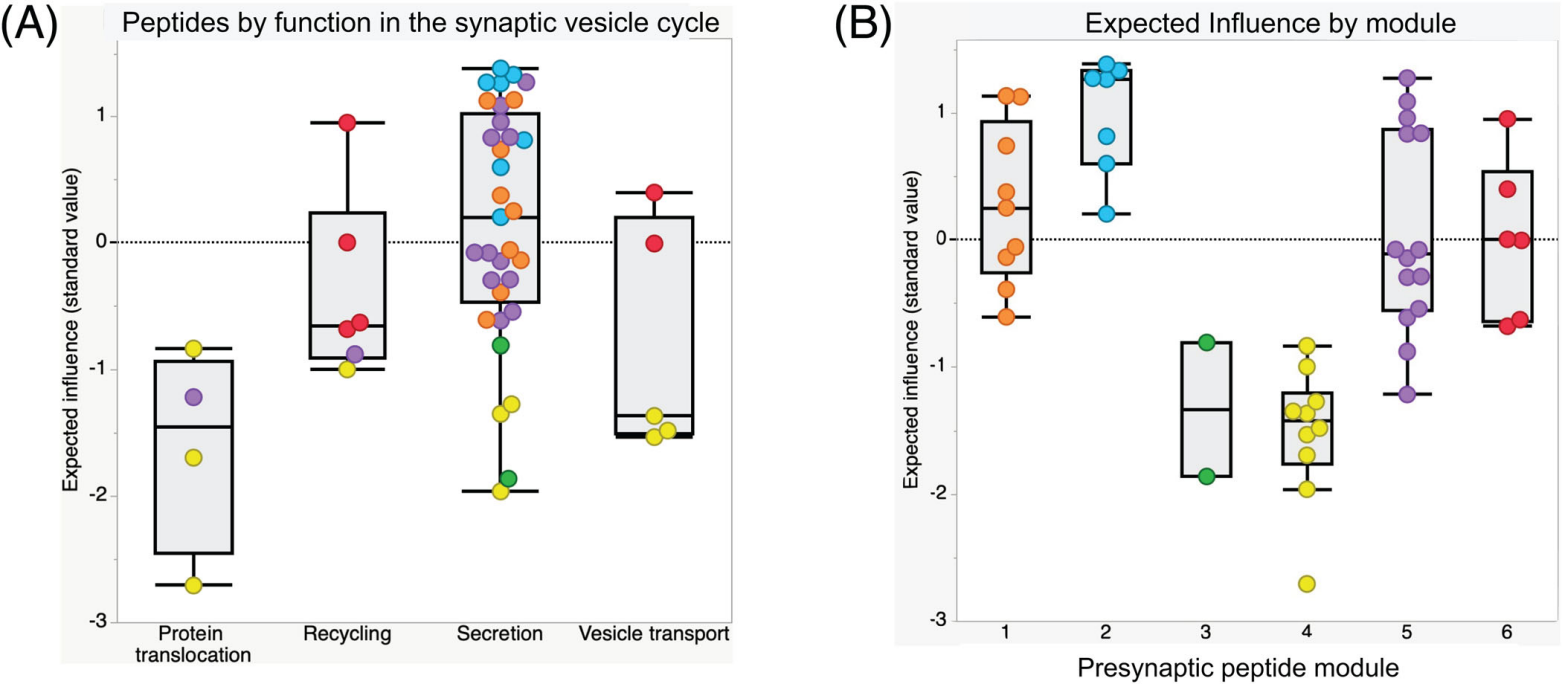
Boxplots of expected influence values of synaptic peptide nodes according to (A) the main function in the synaptic vesicle cycle and (B) the module membership.

To understand which modules of the synaptic network may be the most influential among the nodes of the multilayer network, we examined expected influence scores within the multilayer network. The mean expected influence score for nodes differed according to functional groupings (F = 5.60, *P* = 0.002, Figure 4A). Synaptic peptide nodes directly involved in secretory function evidenced the highest expected influence within the multilayer network. The six synaptic modules also evidenced differences in their mean expected influence on the multilayer network (F = 15.71, *P* < 0.0001, Figure 4B). Module 2 demonstrated the highest mean expected influence score, with all nodes in this module having a secretory function.

Last, to evaluate potential clinical relevance of the identified synaptic modules, the association between mean module levels with global cognitive performance was analyzed, adjusting for age, sex, and education (Table 2). Higher mean peptide levels in synaptic module 2 were also most strongly associated with better global cognitive performances before death in older adults. After correction for multiple testing, no other module showed a statistically significant association with global cognition.

**TABLE 2 alz14286-tbl-0002:** Association of synaptic peptide modules with global cognitive function nearest to death. Each of the six separate analyses of variance are controlled for age, sex, and education (*n* = 440).

	Estimate	Std error	*t* ratio	*p* value
Module 1	0.55	0.24	2.34	0.02
Module 2	1.68	0.29	5.85	<0.0001
Module 3	−0.05	0.20	−0.25	0.81
Module 4	−0.54	0.35	−1.52	0.13
Module 5	0.15	0.27	0.54	0.59
Module 6	0.97	0.70	1.39	0.17

Together, these findings suggest that synaptic peptides directly involved in neuronal secretory function, particularly those represented in module 2, are both the most influential in the multilayer network and evidence the strongest associations with cognitive performance.

### 3.4 Multilayer network properties in higher versus lower physical activity groups

**FIGURE 5 alz14286-fig-0005:**
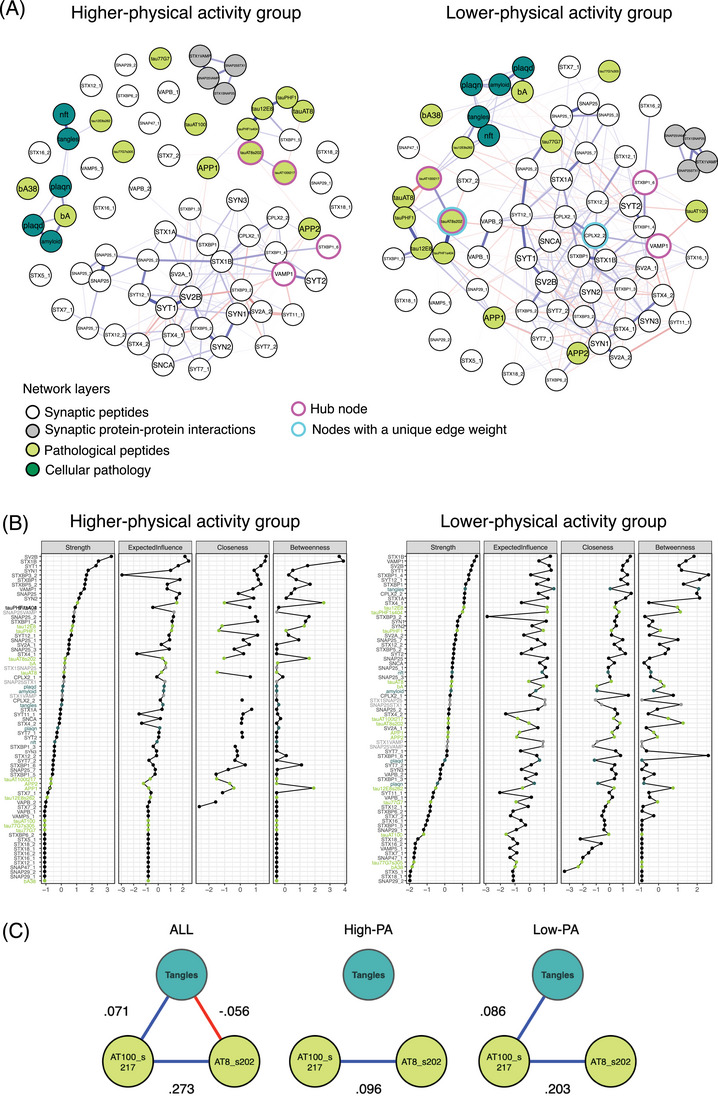
A, Multilayer network of synaptic peptides (white), synaptic protein–protein interactions (gray), pathological peptides (lime), and cellular pathology (teal) for the higher physical activity group versus lower physical activity group. Hub nodes highlighted in pink. Nodes with an edge unique to the lower physical activity identified by bootstrap permutation testing are highlighted in cyan. Edge weights represent the positive (blue) and negative (red) conditional dependencies between nodes. Fruchterman–Reingold layout whereby nodes closely positioned have stronger relationships. B, Centrality measures (*z* scores) for all synaptic peptides (black), synaptic protein–protein interactions (gray), pathological peptides (lime), and cellular pathology (teal) nodes of the multilayer network. C, Schematic figure demonstrating the unbalanced triangle including two hub nodes in the multilayer network. Values are edge weights, with blue representing positive edges and red representing negative edges. High‐PA, higher physical activity group; Low‐PA, lower physical activity group.

To understand how a modifiable lifestyle behavior associated with both AD risk and synaptic health may affect network properties, the multilayer network structure was examined in the sample as a whole, and in groups with higher versus lower physical activity based on actigraphy monitoring (Figure 5A,B). The following properties of network structure were analyzed: global connectivity, individual edge strengths between nodes—compared between higher and lower physical activity groups, and higher order organizational structure by examining triadic relationships—nodes linked in a triangular geometry.

Global connectivity was greater in the lower physical activity group (higher physical activity 13.144, lower physical activity 26.224, *P* = 0.186 by bootstrap testing, Table S5 in supporting information). In particular, the largest difference in inter‐layer connectivity was between the synaptic peptide to pathologic peptide layers (higher physical activity 0.202, lower physical activity 1.422, Table S5). There were also structural differences in pairwise nodal associations between groups. Bootstrap permutation testing[Table alz14286-tbl-0002] indicated the negative edge between the synaptic peptide CPLX2_2 and phosphorylated tau_AT8_s202 was unique to the lower physical activity group (*P* < 0.001, Holm–Bonferroni correction for multiple comparisons).

In addition to pairwise connections, the higher order organizational structural balance of triadic interactions was explored. There was a high degree (0.800) of global structural balance (the fraction of balanced triangles^58^) in the overall multilayer network. This indicates that, as with other biological networks, this multilayer network is inclined to assume a more stable configuration. Of note, an unbalanced triangle motif emerged within the multilayer network between the levels of phosphorylated isoforms of tau (tau_AT100_t217 and tau_AT8_s202 hub nodes) and the level of cellular tangle pathology, illustrated in Figure 5C. Unbalanced triangles represent a source of tension in network structural dynamics, whereby these relationships may be tenuous, and potentially amenable to change.^57^, ^58^ Indeed, considering the higher and lower physical activity groups separately helps resolve the origins of the imbalance. In the higher physical activity group, cellular tangle pathology was not associated with either of these isoforms, while in the lower physical activity group a positive association between tau_AT100_s217 and tangle pathology was observed.[Fig alz14286-fig-0005]

### 3.5 Network‐informed analyses of physical activity alongside synaptic and tangle burden

The pattern of results from the multilayer network suggests associations among synaptic peptides, pathological peptides, and cellular pathology differ according to level of physical activity. Specifically, associations between phosphorylated isoforms of tau (tau_AT100_t217 and tau_AT8_s202) and cellular tangle pathology were stronger in lower versus higher physical activity adults. The expected influence of synaptic peptide module 2 on the multilayer network, as well as the functional characteristics of nodes in this module and the unique association with cognition suggest it may be a particularly relevant module to examine more deeply. A series of models were therefore constructed to evaluate the role of physical activity (modeled as continuous daily counts) on relationships between synaptic module 2 and identified pathologic tau peptides (tau_AT100_t217 and tau_AT8_s202) on tangle burden.

The effects of three‐way interactions between each phosphorylated tau isoform (tau_AT100_t217 or tau_AT8_s202), synaptic module 2, and physical activity levels, on cellular tangle pathology burden were tested in the same model (Table S6 in supporting information). As expected, tau_AT100_t217 showed a positive association with tangle burden (beta 1.08, standard error [SE] 0.06, *P* < 0.0001), while the association with tau_AT8_s202 was negative (beta −0.97, SE 0.11, *P* < 0.0001). Higher levels of module 2 synaptic peptides were associated with lower tangle burden (beta −1.11 m, SE 0.27, *P* < 0.0001). Further, there was a three‐way interaction with physical activity, such that in individuals with lower levels of physical activity and low levels of synaptic module 2 peptides the relationship between tau_AT100_t217 and tangle burden was the largest (beta 0.65, SE 0.33, *P* = 0.0462). In other words, those individuals preserving key components of the neurosecretion machinery and with more active lifestyles may be more resilient to the conversion of p‐tau into tangle pathology.

### 3.6 A summative exploration of the four‐level network in the lower physical activity group

**FIGURE 6:**
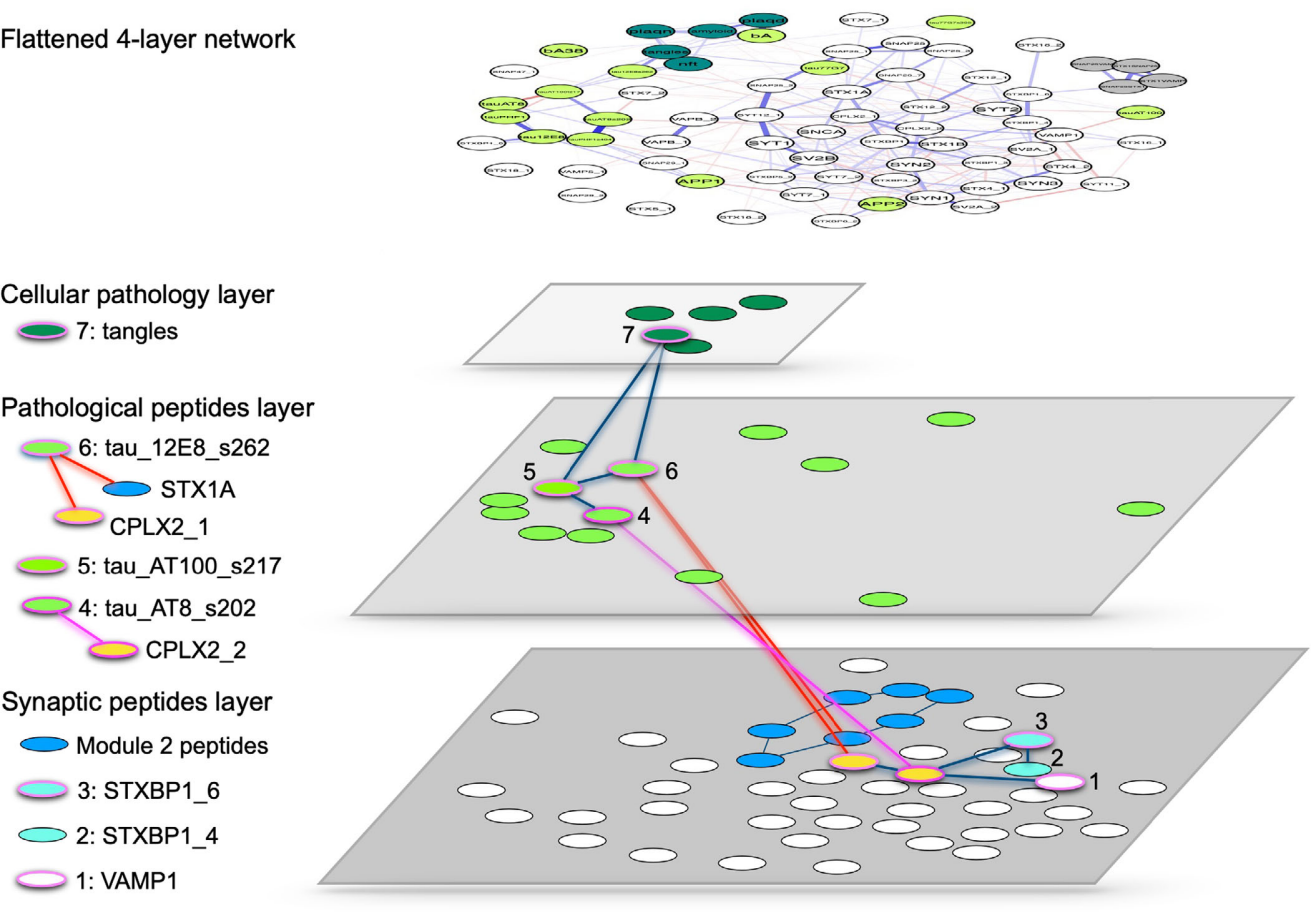
Schematic depiction of multilayer network among older adults with lower physical activity, demonstrating the cellular pathology (teal), pathological peptide (lime), and synaptic peptide (white) layers, as well as the module 1 peptides (orange), module 2 peptides (blue), and hub nodes (highlighted in pink). Putative pathways for neurodegeneration are highlighted in red, and an edge unique to the lower physical activity group is highlighted in pink.

Multilevel network models allow visualization of possible pathways relevant to neurodegeneration (Figure 6). The negative valence edge connecting the pathological peptide hub node tau_AT8_s202 to the complexin‐2 peptide CPLX2_2 indicates a pathway for neurodegeneration unique to the lower physical activity group. Within the synaptic layer, the CPLX2_2 node is further connected to hub nodes STXBP1_6 and VAMP1. Within the pathological peptide layer, the hub node tau_AT8_s202 also connects positively with the hub node tau_AT100_s217, which in turn connects to the tangle's node of the cellular pathology layer via tau_12E8_s262. The tau_12E8_s262 node is also compelling as it provides two additional routes for neurodegeneration. The first is through a negative connection to the second complexin‐2 peptide (CPLX2_1) linking with the tau_AT8_s202 pathway. The second is through a negative connection to the syntaxin‐1A node (STX1A), part of synaptic module 2, described above as being the module most strongly associated with cognitive functioning. These putative pathways for neurodegeneration suggest how lower physical activity may create vulnerability for the effects of tau phosphorylation in the proline‐rich domain on tangle formation, and on synaptic peptides involved in secretion and related to cognition.

## 4 DISCUSSION

The present study characterized the relationships among brain tissue synaptic peptides, AD pathological peptides, and cellular pathology, and late‐life physical activity using a multilayer network analysis approach in community dwelling older adults. This approach identified distinct pathological and synaptic peptides as network hubs, and revealed that synaptic peptides directly involved in neuronal secretory function were the most influential contributors to the complex interdependencies between AD pathology and synaptic processes. Among the synaptic peptides, again those with specific secretory functions further showed the strongest associations with late‐life cognitive performance, underscoring their clinical relevance. When grouped according to higher or lower physical activity level, vulnerability of the synaptic peptide layer to neurodegenerative influences from tangles and phosphorylated tau peptides, particularly involving the proline‐rich domain, was apparent. Interestingly, nodes involving amyloid were not identified as key influential factors in the multilayer network. This study both highlights the analytic utility of network‐based approaches to model interdependent but unique biological processes simultaneously, and advances the conceptual understanding of possible cellular mechanisms underlying the role of physical activity in modifying the risk of neurodegeneration.

Inclusion of the multilayer datasets comprised synaptic and AD peptides, and cellular pathology advances our understanding toward a more complete overview of the interactive biological mechanisms underlying brain physiology in late life. Consistent with other network studies of neurodegeneration,^66^, ^67^ AD neuropathology layers showed direct relationships with individual synaptic peptide nodes as well as communities of synaptic peptides (“modules”). Examining the larger proteome, a recent multilayer network study isolated a strong negative relationship between synaptic protein modules and tau microtubule‐binding domain peptide levels.^42^ Modules were co‐located with Aβ plaques and neurofibrillary tangles and associated with global cognition. The present multilayer network study complements this work by focusing on 48 synaptic peptides involved in critical presynaptic machinery functions previously shown to be relevant to cognition.^12^ A modular architecture was observed within the synaptic peptide layer. The module including SNAP‐25, syntaxin‐1A, and synaptotagmin‐12 peptides showed greatest influence on other network nodes, and a positive association with cognition. No other module had a statistically significant association with global cognition. Independent analyses of these peptides in the same samples demonstrated associations of syntaxin‐1A and synaptotagmin‐12 with motor resilience.^68^

Synaptic peptides with specific roles in neurosecretion had the strongest relationship with late‐life cognitive performance. Indeed, aberrant soluble N‐ethylmaleimide‐sensitive factor attachment protein receptor protein–protein (SNARE)‐mediated secretion, facilitated by SNAP25, STX1, and VAMP, is linked with multiple neurological diseases with cognitive impairment as a core feature, including encephalopathy, schizophrenia, and dementia.^45^ Levels of these peptides were also the most influential to the network structure, indicating that modification of these peptides in particular may have significant downstream consequences on the relationships between pathological and synaptic peptides. Thus, aberrant synaptic transmission may be an early step in the pathway of neuropathology‐driven synapse dysfunction and loss.^12^ Understanding the threshold for dysfunction may be an important area for future investigation.

Dementia, including AD, may have a long prodromal period prior to irreversible progressive cognitive decline. Curtailing this decline with accessible interventions is an important public health priority. Thus, identifying the neurobiology of physical activity is needed to both enhance precision of dementia prevention strategies and facilitate identification of possible treatment targets underlying known neuroprotective behaviors. The present results suggest that physical activity may buttress brain reserve by weakening the influence of neuropathologies, namely pathological tau, on the synaptic proteome. Three findings related to between‐layer network connections are of note. First, the unbalanced triangle in the overall network formed by tangles, p‐tau s217, and p‐tau s202 was resolved when separate networks were mapped for the higher and lower physical activity groups. In the higher physical activity group, no statistically significant edges were detected between tangles and these two p‐tau peptide hubs derived from the proline‐rich region of tau. In contrast, positive edges were observed between the two p‐tau peptides, and between p‐tau s217 and tangles in the lower physical activity group. Second, uniquely in the lower physical activity group, greater levels of p‐tau s202 were associated with lower levels of the synaptic peptide CPLX2_2. This peptide is derived from complexin‐2, found in excitatory (glutamatergic) terminals, and vulnerable in the later Braak stages V–VI of AD.^69^ The CPLX2_2 node is linked directly to the hub nodes VAMP1 and STXBP1_6. The latter is derived from the isoform of the synaptic protein known as Munc‐18‐1, a syntaxin binding protein found in inhibitory (GABAergic) terminals—also vulnerable in the later Braak stages of AD.^65^ Together, this suggests a close link between p‐tau and both excitatory and inhibitory synaptic dysfunction only in lower activity older adults. The third finding is the connections detected among p‐tau s262, tangles, and two synaptic peptide nodes in the lower physical activity group. This p‐tau node maps to the microtubule binding domain of tau, shows a strong connection to the tangles node in the cellular pathology layer, and although not modeled here, is reported to be associated with prefrontal anterior watershed region due to small vessel disease.^42^ In the multi‐level network, higher levels of p‐tau 262 are associated with lower levels of CPLX2_1, derived from complexin‐2, and with lower levels of STX1A, the peptide marker node for synatxin‐1A. Syntaxin‐1A is highly enriched in excitatory terminals; however, it shows vulnerability to loss in mild cognitive impairment as well as AD dementia.^12^ In the higher physical activity group, the p‐tau s262 node was linked to tangles in the cellular pathology layer, but had no statistically significant connections to the synaptic peptide layer.

These findings suggest that participants with higher physical activity in their later years have weaker connections between molecular and cellular manifestations of pathology, and are less vulnerable to neurodegeneration associated with p‐tau effects on synapses. Decades of animal studies causally link physical activity to synaptogenesis,^70^, ^71^ a relationship that is beginning to be recapitulated in human studies.^21^, ^28^ However, while some animal studies suggest exercise induces reductions in pathogenic tau burden,^72^, ^73^ observational and limited intervention studies in humans are more mixed.^23^, ^74^, ^75^ Instead, other human studies suggest that physical activity may *moderate* the association between tau and clinical manifestation of impairment.^23^, ^76^ These latter data may be consistent with the current study showing that higher daily physical activity relates to decreases in the magnitude of relationships between p‐tau peptides and synaptic and tangle pathology biomarkers.

Acquisition of such rich information was made possible due to high‐quality neuropathologic, immunohistochemistry, protein–protein interaction analysis, and the optimized selected reaction monitoring mass spectrometry‐based proteomics, in addition to extensive cognitive and actigraphy measures. Nonetheless, there are several limitations of our study. The observational nature of these data preclude ability to determine causality; there are likely bidirectional relationships particularly between physical activity and synaptic and AD pathogenic processes. Nonetheless, these data help identify clinically relevant targets for further mechanistic study. Additionally, although the synaptic peptides were selected based on their relevance in SNARE‐mediated exocytosis, as well as their potential role in AD pathogenesis, they do not represent the full synaptic proteome, particularly postsynaptic processes. Future works integrating continued advancements in ‐omics technology will provide more in‐depth molecular windows into these brain–behavior processes. Given the synaptic and pathology indicators were quantified in brain tissue at autopsy, the dynamic temporality of the relationships, particularly between physical activity and proteomic outcomes, cannot be captured. Indeed, earlier life activity patterns may be related to late‐life activity and proteomic outcomes, though late‐life physical activity is most consistently associated with dementia risk, memory performance, and plaque burden.^77^, ^78^ Further, given AD is the most common dementia etiology and the pathology most strongly associated with cognitive decline in late life, we focused the current network analysis on understanding AD‐related relationships, including availability of a panel of AD‐related tau isoforms. However, future work incorporating relevant peptides representing the range of common age‐related neuropathologies is needed for a fully comprehensive understanding. Peptide analyses were quantified from the DLPFC in this study; given the high localization properties of the brain, relationships among synaptic, behavioral, and pathological physiology may differ across brain regions. However, of note, our prior work suggests that relationships between physical activity and molecular synaptic biomarkers did not depend on region of the brain assayed.^28^ Last, our sample was also largely White with greater than a high school education, which may not generalize to other communities; incorporation of diverse representation in autopsy studies is currently limited, and urgently needed to identify effective biomarkers and intervention points for the broader population.

In sum, our novel approach incorporating proteomic and behavioral data into multilayer network analyses highlights both the utility of advanced approaches to high dimensional biobehavioral data and underscores new insights into the interactions among the synaptic proteome, AD pathophysiology, and lifestyle behavior. Synaptic secretory processes were particularly important in the context of AD pathogenesis biomarkers and may represent targets with high levels of influence. Further, physical activity may weaken pathogenic relationships among secretion, p‐tau, and tangle pathology in older adults.

## CONFLICT OF INTEREST STATEMENT

William G. Honer has received consulting fees or sat on paid advisory boards for: Translational Life Sciences, AbbVie, Boehringer Ingelheim, and Newron. The organizations cited above had no role in (and therefore did not influence) the design of the present study, the interpretation of results, and/or preparation of the manuscript. Andrea A. Jones, Alfredo Ramos‐Miguel, Kristina M. Gicas, Vladislav A. Petyuk, Sue E. Leurgans, Philip L. De Jager, Julie A. Schneider, David A. Bennett, and Kaitlin B. Casaletto have no potential conflict of interests. Author disclosures are available in the supporting information.

## CONSENT STATEMENT

Informed consent was provided by all subjects for clinical assessment, repository, and autopsy, allowing data and specimens to be shared for research studies.

## Supporting information



Supporting Information

Supporting Information
