# Supplementary material for: A multilayer network analysis of Alzheimer's disease pathogenesis: Roles for p‐tau, synaptic peptides, and physical activity
Source: Alzheimers Dement. 2024 Oct 12;20(11):8012–27. doi: 10.1002/alz.14286 (PMC11567865; doi:10.1002/alz.14286)
Supplement: Supplementary file 1 — Supporting Information [file ALZ-20-8012-s002.docx]

**Supplemental Materials**

**Supplemental Figure S1.** Centrality measures (z-scores) presented for nodes of multilayer network.

**Supplemental Table S1.** Synaptic peptide layer

| Node | Peptide ID | Peptide sequence | Comm | Target | Main function | Subcellular localisation | Cell type | Notes |
| --- | --- | --- | --- | --- | --- | --- | --- | --- |
| CPLX2_1 | CPLX2_1 | AALEQPCEGSLTRPK | 1 | Complexin-2 | Secretion | Cytosol | Neuron |  |
| CPLX2_2 | CPLX2_2 | YLPGPLQDMFK |  |  |  |  |  |  |
| STX1B | STX1B_1 | QHSAILAAPNPDEK | 1 | Syntaxin-1B | Secretion | P-membrane | Neuron | Isoform specific, glutamate and GABA |
|  | STX1B_2 | AIEQSIEQEEGLNR |  |  |  |  |  |  |
| STXBP1 | STXBP1_1 | SSASFSTTAVSAR | 1 | Syntaxin-binding protein 1 | Secretion | Cytosol | Neuron | Not isoform-specific, binds to syntaxin |
|  | STXBP1_2 | HIAEVSQEVTR |  |  |  |  |  |  |
| STXBP1_4 | STXBP1_4 | WEVLIGSTHILTPTK | 1 | Syntaxin-binding protein 1 | Secretion | Cytosol | Neuron | Isoform 1 (long variant). GABA specific |
| STXBP1_6 | STXBP1_6 | VSFEDQAPTME |  |  |  |  |  | Isoform 1 (long variant). GABA specific |
| SYN3 | SYN3_1 | SFKPDFILVR | 1 | Synapsin-3 | Secretion | V-membrane | Neuron |  |
|  | SYN3_2 | SQSLTNSLSTSDTSQR |  |  |  |  |  |  |
| SYT2 | SYT2_1 | IFVGSNATGTELR | 1 | Synaptotagmin-2 | Secretion | V-membrane | Neuron | Fast calcium sensor |
|  | SYT2_2 | LTVCILEAK |  |  |  |  |  |  |
| VAMP1 | VAMP1_1 | ADALQAGASQFESSAAK | 1 | Vesicle-associated membrane protein 1 | Secretion | V-membrane | Neuron |  |
|  | VAMP1_2 | LQQTQAQVEEVVDIIR |  |  |  |  |  |  |
| SNAP25_1 | SNAP25_1 | HMALDMGNEIDTQNR | 2 | Synaptosomal-associated protein 25 | Secretion | P-membrane | Neuron | Excluded in SNAP25 N-terminal fragment |
| SNAP25_2 | SNAP25_2 | AWGNNQDGVVASQPAR | 2 |  |  |  |  | Likely contains SNAP25 cleavage site |
| SNAP25_3 | SNAP25_3 | ADQLADESLESTR | 2 |  |  |  |  | Included in SNAP25 N-terminal fragment |
| SNAP25 | SNAP25_4 | TLVMLDEQGEQLER | 2 |  |  |  |  | SNAP25B (1 aa different in this sequence from SNAP25A), more stable SNARE complex |
|  | SNAP25_6 | FCGLCVCPCNK |  |  |  |  |  |  |
| SNAP25_7 | SNAP25_7 | CCGLFICPCNK | 2 |  |  |  |  | SNAP25A splice variant |
| STX1A | STX1A_1 | SIEQSIEQEEGLNR | 2 | Syntaxin-1A | Secretion | P-membrane | Neuron | isoform specific, neuronal, mainly glutamatergic |
|  | STX1A_2 | QALSEIETR |  |  |  |  |  |  |
| SYT12_1 | SYT12_1 | VSLLPDEQIVGISR | 2 | Synaptotagmin-12 | Secretion | V-membrane | Neuron |  |
| SNAP29_1 | SNAP29_1 | DLPDGPDAPADR | 3 | Synaptosomal-associated protein 29 | Secretion | P-membrane | Ubiquitous |  |
| VAMP5_1 | VAMP5_1 | SDQLLDMSSTFNK | 3 | Vesicle-associated membrane protein 5 | Secretion | V-membrane | Neuron |  |
| SNAP29_2 | SNAP29_2 | SVFGGLVNYFK | 4 | Synaptosomal-associated protein 29 | Secretion | P-membrane | Ubiquitous |  |
| STX4_1 | STX4_1 | NILSSADYVER | 4 | Syntaxin-4 | Protein trans-location | P-membrane | Ubiquitous |  |
| STX4_2 | STX4_2 | HSEIQQLER | 4 |  |  |  |  |  |
| STX5_1 | STX5_1 | HLQTHSNTIVVSLQSK | 4 | Syntaxin-5 | V-transport | ER/Golgi | Ubiquitous |  |
| STX16_2 | STX16_2 | LTDAFLLLR | 4 | Syntaxin-16 | Recycling | ER/Golgi | Ubiquitous |  |
| STX18_1 | STX18_1 | TAVLDFIEDYLK | 4 | Syntaxin-18 | Vesicle transport | ER/Golgi | Ubiquitous |  |
| STX18_2 | STX18_2 | TCSEAIQQLR | 4 |  |  |  |  |  |
| STXBP3_2 | STXBP3_2 | AAYIYFTDFCPDNLFNK | 4 | Syntaxin-binding protein 3 | Protein trans-location | Cytosol | Neuron |  |
| STXBP6_2 | STXBP6_2 | QFEGSTSFVR | 4 | Syntaxin-binding protein 6 | Secretion | P-membrane | Neuron |  |
| SYT11_1 | SYT11_1 | NLLVDAAEAGLLSR | 4 | Synaptotagmin-11 | Secretion | V-membrane | Neuron |  |
| SNAP47_1 | SNAP47_1 | NVVFSIIEHFWR | 5 | Synaptosomal-associated protein 47 | Protein trans-location | P-membrane | Ubiquitous |  |
| SNCA | SNCA_1 | EGVVHGVATVAEK | 5 | Alpha-synuclein | Secretion | V-membrane | Neuron | May promote trimeric SNARE complex formation, bind to VAMP2 |
|  | SNCA_2 | TVEGAGSIAAATGFVK |  |  |  |  |  |  |
| STX16_1 | STX16_1 | IDYNVEQSCIK | 5 | Syntaxin-16 | Recycling | ER/Golgi | Ubiquitous |  |
| STXBP1_3 | STXBP1_3 | WEVLIGSTHILTPQK | 5 | Syntaxin-binding protein 1 | Secretion | Cytosol | Neuron | Isoform 2 (short variant). ubiquitous |
| STXBP1_5 | STXBP1_5 | TDEEISS | 5 | Syntaxin-binding protein 1 | Secretion | Cytosol | Neuron | Isoform 2 (short variant) ubiquitous |
| STXBP5_2 | STXBP5_2 | LLQPVIVSPSGTILR | 5 | Syntaxin-binding protein 5 | Secretion | P-membrane | Neuron |  |
| SV2A_1 | SV2A_1 | FEEEDDDDDFPAPSDGYYR | 5 | Synaptic vesicle glycoprotein 2A | Secretion | V-membrane | Neuron |  |
| SV2A_2 | SV2A_2 | GGLSDGEGPPGGR |  |  |  |  |  |  |
| SV2B | SV2B_1 | ATAFGILNGLCK | 5 | Synaptic vesicle glycoprotein 2B | Secretion | V-membrane | Neuron | In some glutamate terminals |
|  | SV2B_2 | VFTVSNIK |  |  |  |  |  |  |
| SYN1 | SYN1_1 | QGPPQKPPGPAGPTR | 5 | Synapsin-1 | Secretion | V-membrane | Neuron |  |
|  | SYN1_2 | LGTEEFPLIDQTFYPNHK |  |  |  |  |  |  |
| SYN2 | SYN2_1 | SFRPDFVLIR | 5 | Synapsin-2 | Secretion | V-membrane | Neuron |  |
|  | SYN2_2 | VLLVVDEPHADWAK |  |  |  |  |  |  |
| SYT1 | SYT1_1 | VFVGYNSTGAELR | 5 | Synaptotagmin-1 | Secretion | V-membrane | Neuron | Fast calcium sensor |
|  | SYT1_2 | LTVVILEAK |  |  |  |  |  |  |
| SYT7_1 | SYT7_1 | NLNPIFNESFAFDIPTEK | 5 | Synaptotagmin-7 | Secretion | V-membrane | Neuron |  |
| SYT7_2 | SYT7_2 | NSLETVGTPDSGR |  |  |  |  |  |  |
| STX7_1 | STX7_1 | TLNQLGTPQDSPELR | 6 | Syntaxin-7 | Recycling | Endosomes | Ubiquitous |  |
| STX7_2 | STX7_2 | EFGSLPTTPSEQR |  |  |  |  |  |  |
| STX12_1 | STX12_1 | LMNDFSAALNNFQAVQR | 6 | Syntaxin-12 | Recycling | Endosomes | Ubiquitous |  |
| STX12_2 | STX12_2 | ELGSLPLPLSTSEQR |  |  |  |  |  |  |
| VAPB_1 | VAPB_1 | EAKPEDLMDSK | 6 | VAMP-associated protein B/C | Vesicle transport | ER/Golgi | Neuron |  |
| VAPB_2 | VAPB_2 | VEQVLSLEPQHELK | 6 |  |  |  |  |  |

**Supplemental Table S2.** Synaptic protein-protein interactions layer

| Node | Target |
| --- | --- |
| SNAP25STX1 | SNAP-25 (bait) – syntaxin-1 interaction in dorsolateral prefrontal cortex |
| STX1SNAP25 | Syntaxin-1 (bait) – SNAP-25 interaction |
| SNAP25VAMP | SNAP-25 (bait) – VAMP interaction |
| STX1VAMP | Syntaxin-1 (bait) – VAMP interaction |

**Supplemental Table S3.** Pathology peptides layer

| Node | Peptide ID | Peptide sequence | Target |
| --- | --- | --- | --- |
| tau_AT8 | MAPT_1 | SGYSSPGSPGTPGSR | Peptide from tau recognised by antibody AT8 when phosphorylated |
| tau_AT8_s202 | MAPT_2 | SGYSSPG**S**PGTPGSR | Tau peptide with serine 202 phosphorylation |
| tau_AT100 | MAPT_18 | TPSLPTPPTREPK | Peptide from tau recognised by antibody AT100 when phosphorylated |
| tau_AT100_t217 | MAPT_19 | TPSLP**T**PPTREPK | Tau peptide with threonine 217 phosphorylation |
| tau_12E8 | MAPT_28 | IGSTENLK | Peptide from tau recognised by antibody 12E8 when phosphorylated |
| tau_12E8_s262 | MAPT_29 | IG**S**TENLK | Tau peptide with serine 262 phosphorylation |
| tau_77G7 | MAPT_35 | HVPGGGSVQIVYKPVDLSK | Peptide from tau recognised by antibody 77G7 when phosphorylated |
| tau_77G7_s305 | MAPT_36 | HVPGGG**S**VQIVYKPVDLSK | Tau peptide with serine 305 phosphorylation |
| tau_PHF1 | MAPT_47 | SPVVSGDTSPR | Peptide from tau recognised by antibody PHF1 when phosphorylated |
| tau_PHF1_s404 | MAPT_48 | SPVVSGDT**S**PR | Tau peptide with serine 404 phosphorylation |
| APP1 | APP_1 | EVCSEQAETGPCR | Amyloid-beta precursor protein aa 233-245 |
| APP2 | APP_2 | THPHFVIPYR | Amyloid-beta precursor protein aa 51-60 |
| bA | APP_3 | LVFFAEDVGSNK | Amyloid-beta |
| bA38 | APP_5 | GAIIGLMVGG | Amyloid-beta-38 |

**Supplemental Table S4.** Cellular pathology layer

| Node | Target |
| --- | --- |
| plaqd | Diffuse plaque density in middle frontal cortex identified with Bielschowsky staining |
| plaqn | Neuritic plaque density in middle frontal cortex identified with Bielschowsky staining |
| amyloid | Percent area of microscopic field occupied by immunoreactive amyloid-beta |
| nft | Neurofibrillary tangle density in middle frontal cortex identified with Bielschowsky staining |
| tangles | Neurofibrillary tangle density in middle frontal cortex identified with AT8 immunostaining |

|  | **Whole Sample** | **High PA** | **Low PA** |
| --- | --- | --- | --- |
| Global Connectivity | 32.770 | 13.144 | 26.224 |
| Intra-Layer Connectivity |  |  |  |
| Synaptic Peptide Layer | 19.118 | 9.260 | 15.990 |
| Synaptic Protein-Protein Complexes Layer | 1.655 | 0.962 | 1.587 |
| Pathological Peptide Layer | 3.736 | 1.417 | 2.660 |
| Cellular Pathology Layer | 1.503 | 0.597 | 1.264 |
| Inter-Layer Connectivity |  |  |  |
| Synaptic Peptide to P-P Complex | 0.039 | 0 | 0.053 |
| Synaptic Peptide to Pathological Peptide | 2.373 | 0.202 | 1.442 |
| Synaptic Peptide to Cellular Pathology | 0.136 | 0 | 0.133 |
| Synaptic P-P Complex to Pathological Peptide | 0.021 | 0 | 0 |
| Synaptic P-P Complex to Cellular Pathology | 0 | 0 | 0 |
| Pathological Peptide to Cellular Pathology | 0.812 | 0.252 | 0.734 |

**Supplemental Table S5**

**Supplemental Table S6.**

|  | Estimate | Std Error | t Ratio | p-value |
| --- | --- | --- | --- | --- |
| Intercept | 1.21596657 | 0.73597645 | 1.65 | 0.0992 |
| age_death | -0.01014 | 0.00764417 | -1.33 | 0.1854 |
| educ | -0.0046046 | 0.01646261 | -0.28 | 0.7798 |
| msex | -0.2560725 | 0.10519761 | -2.43 | 0.0153 |
| amyloid_mf_sqrt | 0.15240541 | 0.03511745 | 4.34 | <.0001 |
| tau_AT8_s202 | -0.967179 | 0.1121449 | -8.62 | <.0001 |
| tau_AT100_t217 | 1.08308174 | 0.06101752 | 17.75 | <.0001 |
| average_daily | 0.04905283 | 0.04057477 | 1.21 | 0.2274 |
| synaptic module-2 | -1.1135756 | 0.27151563 | -4.1 | <.0001 |
| (tau_AT8_s202+0.04794)*(synaptic module-2+0.00985) | 0.50784298 | 0.56269496 | 0.9 | 0.3673 |
| (tau_AT100_t217-0.04086)*(synaptic module-2+0.00985) | -1.6055181 | 0.29087526 | -5.52 | <.0001 |
| (tau_AT8_s202+0.04794)*(average_daily-1.94193) | -0.1459528 | 0.116867 | -1.25 | 0.2124 |
| (tau_AT100_t217-0.04086)*(average_daily-1.94193) | 0.00067706 | 0.05303072 | 0.01 | 0.9898 |
| (synaptic module-2+0.00985)*(average_daily-1.94193) | -0.1451722 | 0.25836978 | -0.56 | 0.5745 |
| (tau_AT8_s202+0.04794)*(comm-2+0.00985)*(average_daily-1.94193) | 0.07578901 | 0.70713781 | 0.11 | 0.9147 |
| (tau_AT100_t217-0.04086)*(synaptic module-2+0.00985)*(average_daily-1.94193) | 0.65166466 | 0.3258709 | 2 | 0.0462 |
